# Thyroid Screening During Early Pregnancy and the Need for Trimester Specific Reference Ranges: A Cross-Sectional Study in Lahore, Pakistan

**DOI:** 10.7759/cureus.5661

**Published:** 2019-09-15

**Authors:** Afnan Talat, Aleena A Khan, Samia Nasreen, John A Wass

**Affiliations:** 1 Medicine, Combined Military Hospital Lahore Medical College and Institute of Dentistry, Lahore, PAK; 2 Gynaecology, Combined Military Hospital Lahore Medical College and Institute of Dentistry, Lahore, PAK; 3 Endocrinology, Oxford Centre for Diabetes, Endocrinology and Metabolism, Oxford, GBR

**Keywords:** thyroid dysfunction, lahore, subclinical hypothyroidism, hyperthyroidism, early pregnancy, pakistan, early pregnancy, hypothyroidism, subclinical hyperthyroidism, reference range

## Abstract

Background

Derangements in thyroid hormone levels can cause multiple complications in the mother and the foetus. Thyroid stimulating hormone (TSH) and free thyroxine (free T4 or FT4) levels are used to screen for maternal thyroid dysfunction; these should be compared with population based trimester-specific reference ranges. Our goal was: to determine the prevalence of various thyroid derangements, in early pregnancy, according to the current reference ranges available; to determine the need for trimester specific reference ranges for the local population.

Methods

A multi-centric, cross sectional population survey was conducted in Lahore, Pakistan. Serum TSH and FT4 levels were measured at the hormone lab of the Pathology department of Combined Military Hospital (CMH) Lahore. The results were entered and analysed using Statistical Package for the Social Sciences (SPSS) version 23.

Results

In the 293 women sampled, mean FT4 and TSH levels were 15.03 (±5.62) pmol/L and 2.53 (±6.82) mIU/L respectively. According to the laboratory specific reference ranges, the prevalence of overt hyperthyroidism was 4.10%, (mean TSH= 0.03mIU/L); subclinical hyperthyroidism was 16.38%, (mean TSH= 0.17mIU/L); normal 70.65%, (mean TSH = 1.29mIU/L); subclinical hypothyroidism 4.44%, (mean TSH= 15.11mIU/L); overt hypothyroidism 4.44%, (mean TSH = 20.60mIU/L).

Conclusion

Our study showed a significant prevalence of thyroid dysfunction in the first trimester of pregnancy, and therefore highlights the need for more rigorous thyroid screening of women, in early pregnancy. There is a need to monitor these women in order to reduce maternal and foetal complications. Trimester specific reference ranges for thyroid hormones need to be developed in Pakistan.

## Introduction

Pregnancy induces a great demand on the maternal thyroid gland, as it has to adapt to the multiple physiological changes taking place in the body during this time. It has to produce adequate amounts of thyroid hormone in order to meet the demands of the mother and the fetus, as up till the 12th week of gestation, the fetus is completely dependent on the mother for its supply of thyroid hormones [1]. The fetal thyroid gland begins to produce thyroid hormones by the end of the first trimester but that too, not independently, as it still is in need of an adequate supply of iodine from the mother [2]. In order to meet this increased demand, the thyroid gland undergoes several structural and functional changes, induced by the several physiological mechanisms in the mother’s body explained ahead.

The thyroid gland undergoes several functional changes during pregnancy which include: 1) an increase in the production of total triiodothrionine (T3) and thyroxine (T4) due to the stimulatory effects of beta-human chorionic gonadotropin (β-hCG) and human chorionic thyrotropin (hCT) [3], 2) A decrease in the production of thyroid stimulating hormone (TSH) from the anterior pituitary gland due to the high concentration of β-hCG levels during the first trimester (which can elicit sub-clinical hyperthyroidism) [3-5] and 3) A two to three fold increase in concentrations of thyroid hormone-binding globulin (TBG). This occurs firstly due to a several fold increase in the TBG half-life by estrogen stimulated sialylation of TBG and secondly, due to an increase in hepatic production of TBG [4]. This change further increases the total serum thyroid hormone levels as most of the thyroid hormone (80% of T3 and 68% of T4) circulating in the blood is bound to TBG [4]. 4) The changes to free thyroxine (Free T4 or FT4) and free triiodothrionine (Free T3 or FT3) hormone levels however are a bit complex and remain a point of controversy [3,4].

Some studies report an increase, others report a decrease and a few even report no change in free thyroid hormone levels during the course of a normal pregnancy [4,6-8]. According to few authors, differences in free thyroid hormone levels during pregnancy can also occur due to flaws or variations in the method employed to determine the hormone levels [4,9]. A local study conducted by Elahi et al. on pregnant women in Lahore showed FT4 levels decreased during the first trimester of pregnancy whereas TSH and FT3 values increased [2]. 

The importance of the thyroid hormone can be reflected by the several adverse effects thyroid derangements have on both, the mother and the fetus. In-utero exposure to maternal hypothyroidism is associated with unfavourable outcomes as it increases the chances of intrauterine death, low fetal birth weight, fetal distress and irreversible brain damage in the fetus which manifests as mental retardation, cerebral palsy and poor cognitive development [10,11]. A seven-point intelligent quotient (IQ) deficit was reported among seven to nine year olds born to women with untreated hypothyroidism as compared to children of the same age born to euthyroid mothers [12]. Moreover, women with untreated hypothyroidism during pregnancy also have increased chances of miscarrying, and of developing anemia, preeclampsia, hypertension, cardiac dysfunction, post-partum hemorrhage and placental abruptions [10,11]. Even sub-clinical hypothyroidism has been found to be associated with a higher miscarriage rate, preterm delivery, and a lower IQ of the child [13]. This situation is particularly relevant to the local population since Pakistan has one of the highest reported rates of childhood mental retardation in the world [14]. However, the continuous monitoring of maternal thyroid function and adequately supplementing women in order to maintain their normal thyroid function can reduce the prevalence of most of these complications [15].

Diagnosis of thyroid derangements involves evaluation of thyroid function tests (TFTs). According to the American Thyroid Association (ATA), the ideal test is the serum TSH level that is used along with serum FT4 levels to accurately determine how the gland is functioning [16]. The latest guidelines published by the ATA recommend the use of population-based and trimester-specific reference ranges to accurately categorize and treat pregnant women with thyroid derangements. It is recommended to use an upper limit of 4.0mIU/L for TSH during pregnancy when these specific guidelines are not available [16]. This is a significant change from the previous guidelines which recommended an upper limit of 2.5mIU/L [17]. However, in Pakistan, pregnant women are evaluated according to the internationally used reference ranges as trimester based reference ranges and treatment guidelines specific to the local population have not been developed [18]. Accurate evaluation of thyroid function during pregnancy is crucial for initiation of hormone therapy in women recently diagnosed with thyroid derangements, and for dose adjustment in patients already on hormone therapy. It is known that there is a decrease in the upper and lower limit of maternal TSH relative to the non-pregnant TSH reference range, this decrease being the largest in the first trimester. There is a need for proper evaluation of local data to quantify this decrease for the establishment of trimester specific reference intervals for the Pakistani population.

Thyroid disorders are the second most common endocrine problem encountered during pregnancy in the region as they are reported by 1-2% of the women in their reproductive ages [12]. In iodine rich areas, the most common cause of maternal hypothyroidism is Hashimoto's thyroiditis, but in iodine deficient areas it is iodine deficiencies [19]. Goitre can also develop in women with iodine deficiencies and can warrant a chemical testing of their thyroid function [5]. Pakistan is considered to be one of the most iodine deficient countries in the region and according to a study conducted by Elahi et al. on pregnant women in Lahore, 79.5% of the women were iodine deficient (UI <100 µg/L) and 31.5% had clinically visible goitres, predisposing the mother and the neonate to complications associated with thyroid derangements [20-21].

A study conducted on pregnant women visiting a hospital in the city of Lahore reported 14.6% of the women having higher than normal TSH levels and 2.0% as having levels lower than normal [2]. Further evaluation showed 20.9% of women had FT4 levels lower than normal, whereas only 2.0% had levels higher than normal [2]. These findings are in accordance with the iodine levels of the region as decreased thyroid functioning seems to be more common among pregnant women of the region than an up regulation.

The ATA defines maternal hypothyroidism as a maternal TSH concentration elevated beyond the upper limit of the pregnancy specific reference range. The optimal method of treatment is oral administration of levothyroxine (LT4) for these patients and the avoidance of T3 containing preparations as the fetal central nervous system is impermeable to it. The goal of this supplementation is to attain a TSH in the lower half of the trimester specific reference range. Hypothyroid women who are on LT4 therapy prior to pregnancy also require a dose adjustment as soon as the pregnancy is suspected to maintain the optimal TSH levels required during this time (0.1-4.0mU/L) [17]. Women with sub-clinical hypothyroidism require further investigations before the need for LT4 dosing is determined. Adequate supplementation with LT4 is necessary to avoid maternal and fetal complications of maternal hypothyroidism.

One study reports sub-clinical hypothyroidism to be more common than overt hypothyroidism among the pregnant population in South Asia [19]. The prevalence and variation in thyroid derangement among the pregnant population along with the severe complications associated with leaving the condition untreated raises the age-old question of whether there is a need to screen every patient for a thyroid derangement in the region. These concerns are also associated with the high cost of thyroid function tests and the fact that an initial increase in size of the gland is not even physically visible and can only be detected by an ultrasound scan [5]. Nevertheless the complications of leaving maternal hypothyroidism untreated are severe and mostly avoidable. The question for universal screening can be answered by a more thorough evaluation of the problem by conducting a study at several different locations throughout the city.

This study aims to evaluate the current status of thyroid derangements in the city of Lahore by: 1) establishing the prevalence of the various forms of thyroid derangements in the area 2) by determining the number of women already diagnosed with a thyroid derangement and receiving supplementation, 3) by evaluating if women who are already receiving hormone therapy are adequately supplemented, 4) determining if there is a need for more universal thyroid screening of pregnant women in the area and 5) to determine the need for trimester specific reference ranges based on the local population.

## Materials and methods

A cross sectional survey was conducted at three locations in the city of Lahore. The ethical review committee of Combined Military Hospital (CMH) Lahore Medical College approved the study (Reference Number 001/ERC/CMHLMC). Appropriate authorities were contacted at the various hospitals and the sample collection was carried out after they granted written permission. The fieldwork was carried out between May 2016 and March 2017. The sample consisted of a total of 293 women in their first trimester of pregnancy (Up till 13 weeks) visiting the outpatient departments of the Obstetrics & Gynaecology departments at three different hospitals in the city. A consecutive (non-probability) sampling technique was employed. All participants were residing within Lahore at the time of the data collection. Out of these, 231(78.9%) women were sampled at CMH Lahore, 43(14.8%) at Sir Ganga Ram Hospital and 19(6.6%) at Lahore General Hospital. These sites were chosen due to their high patient load and diversity, giving a good representation of the female population of Lahore.

The purpose and procedure of the study was explained to each woman who fulfilled the inclusion criteria. All women agreeing to participate in the study signed a written consent form (available in English and Urdu) after which a proforma was used to record the name, age and obstetric history (gravidity, parity and abortus) of the patient. The patients were questioned for any history of thyroid pathology and use of medication including levothyroxine supplementation was recorded. The dose regimen was noted for patients who were still taking levothyroxine or have taken the supplement in the past. The gestational age was recorded according to the latest ultrasound scan. They were questioned regarding their socioeconomic status which was then recorded under one of the following categories: low Income (≤ PKR 8,500), lower-middle income (≥ PKR 8,501 and ≤ PKR 33,000), upper-middle income (≥ PKR 33,001 and ≤ PKR 102,000) or high-income (> PKR 102,001).

A 5 ml blood sample was drawn, under aseptic conditions, from each of the women for measurement of their serum Free T4 and TSH measurements. Free hormone levels are preferred over total levels as they are more accurate, reflect the biologically active fraction of the hormone and remain unaffected by changes in binding protein concentrations [22]. Total levels also misleadingly show more than normal levels in healthy individuals [12].

The samples were labelled and transferred to the Hormone lab at the Pathology department of CMH Lahore where they were allowed to coagulate at room temperature after which a centrifuge (Rotofix 32A manufactured by Andreas Hettich GmbH & Co. KG) was used for 5 minutes at 4000 RPM/RCF to separate the sera. The sera was placed in cuvettes and stored temporarily at 2°C. Measurement of FT4 and TSH were done by radioimmunoassay (RIA) carried out the same day using commercial kits for Siemens immulite 1000 (manufactured by Seimens Healthcare GmbH).

The data was entered into and analysed using Statistical Package for Social Sciences (SPSS) version 23 for windows. The sample was divided into into five categories of thyroid hormone levels. This was based on the non-pregnant reference values for FT4 and TSH provided by the Hormone Lab at CMH Lahore (FT4: 7-21 pmol/L, TSH: 0.4-4.5 mIU/L), as shown in table 1. Pearson chi-square test was used to compare the relationship between income groups and the categories of thyroid hormone levels. The participants were also informed about their test results.

**Table 1:** Categories of Thyroid Hormone Levels FT4: Free Thyroxine TSH: Thyroid Stimulating Hormone

Category	Reference Range	
	FT4 (pmol/l)	TSH (mIU/L)
Hyperthyroidism	>21.00	<0.40
Subclinical Hyperthyroidism	7.00-21.00	<0.40
Normal	7.00-21.00	0.40-4.50
Subclinical Hypothyroidism	7.00-21.00	>4.50
Hypothyroidism	<7.00	>4.50

## Results

A total of 310 women meeting the criteria were questioned at the Outpatient Departments of the respective hospitals. Two hundred and ninety three women agreed to participate in the study whereas 17 women refused to give consent (response rate: 94.5%). The age of the sample population ranged from 17-40 years (mean 26.75±4.88). The sample was grouped into economic strata according to their monthly incomes; 273 (93.17%) women fell into the lower-middle Income strata, 16 (5.46%) into the low income and 4 (1.37%) into the upper-middle income group. 

There were 65 (22.2%) women who were primigravida and the remaining 228 (77.82%) women have had a total of 2-10 pregnancies (mean 3.10 ± 1.77), with 0-7 live births (mean 1.38 ± 1.31). Out of the total sample, 133 (45.39%) women have had at least one miscarriage with a mean of 0.77 ± 1.08 miscarriages during one of their previous pregnancies.

The mean TSH of the sample was 2.53±6.82 mIU/L with levels ranging from 0.03 to 63.8 mIU/L. The mean FT4 was 15.03 ± 5.62 pmol/L, with values ranging from 0.003 to 75pmol/l. There was no significant association between the income groups and the categories of thyroid hormone levels (p=0.885).

The women were divided into five groups according to the criteria defined in Table 1, where the normal range for TSH is 0.40-4.50 mIU/Land for FT4 is 7.00 - 21.00 pmol/L mIU/L.

Table 2 shows the number of women falling in each of the five groups of thyroid hormone levels, defined according to the criteria given in table 1, and their respective mean TSH and FT4 levels. The reference ranges used are mentioned in table 1 (0.4-4.5 mIU/L for TSH and 7-21 pmol/L for FT4). There were 207 (70.65%) women in the euthyroid category, 12 (4.10%) women in the overt hyperthyroid category, 48 (16.38%) women in the sub-clinical hyperthyroid category, 13 (4.44%) women in the sub-clinical hypothyroid category, and 13 (4.4%) in the overt hypothyroid category. When analyzed according to the latest guidelines published by the ATA in 2017 which recommended an upper TSH limit of 4.0mIU/L, the results showed no change in the numbers in each of the categories.

**Table 2:** Number, Mean and Range of TSH according to Current Thyroid Hormone Status TSH: Thyroid Stimulating Hormone SD: Standard Deviation

Category	n(%)	Mean TSH [±SD] (mIU/L)	TSH Minimum - Maximum (Range) (mIU/L)
Hyperthyroid	12(4.10)	0.03[±0.05]	0.13 - 0.003 (0.13)
Subclinical Hyperthyroid	48 (16.38)	0.17[±0.12]	0.38 - 0.003 (0.39)
Normal	207(70.65)	1.29[±0.80]	4.0 - 0.4 (3.6)
Subclinical Hypothyroid	13(4.44)	15.11[±16.12]	63 - 4.6 (59.20)
Hypothyroid	13(4.44)	20.60[±16.73]	75 - 11.3 (63.70)
Total	293(100.00)	2.53[±6.82]	75 - 0.003 (74.90)

When grouped according to the previous guidelines published by the ATA in 2011 that recommended a normal range of 0.1-2.5mIU/L for TSH, the total number of hypothyroid women rises to 46 women (15.70%) and the number of hyperthyroid women falls to 30 women (10.24%).

There were 12 women who were previously diagnosed with hypothyroidism and were prescribed thyroxine supplements in the past. Ten of these women were still taking thyroxine. Our results showed that seven women out of the 10 were euthyroid taking an average of 129mcg of thyroxine per day and were thus adequately supplemented, two women were still hypothyroid (mean TSH = 3.08 mIU/L), taking on average 75mcg of thyroxine per day, and one was found to have sub-clinical hypothyroidism (TSH = 12.50mIU/L), taking 50mcg of thyroxine per day, indicating inadequate supplementation. Out of the two women who were currently not taking any thyroxine supplementation, one was found to have sub-clinical hyperthyroidism and the other was euthyroid.

From the total sample, four women had a history of hyperthyroidism. One woman was taking 250mcg of propylthiouracil, one had undergone a thyroidectomy, one was previously prescribed Carbimazole at the time of her diagnosis which was discontinued after the confirmation of pregnancy, and one had never received any intervention.

## Discussion

Since Pakistan is considered an iodine deficient country, the Pakistani population, particularly the pregnant women are at risk of developing moderate to severe iodine deficiency with maternal hypothyroidism and fetal consequences [21]. Pakistan began a campaign known as the national Iodine Deficiency Disorders Control Programme (IDDCP) in 1994 to improve the health status of the population. However, a survey in 2001 found only 27.4% of households surveyed to be using Iodized salt [23]. Attributable factors include the cost, local misconceptions relating iodized salt to family planning and accessibility difficulties encountered by rural households.

A previous study conducted on the general Pakistani population reported a prevalence of overt hypothyroidism to be 4.1% whilst prevalence of sub-clinical hypothyroidism was 5.1%, and the prevalence was reportedly higher in the female population [24]. In our study, 29.4% of the sample population falls out of the normal thyroid reference range. Twenty-six women (8.88%) were diagnosed with hypothyroidism, 13 (4.44%) of whom were overt hypothyroid and 13 (4.44%) were sub-clinically hypothyroid. In contrast, a study conducted in northern India on women in their first trimester of pregnancy reported 143 out of 1000 women (14.3%), as having hypothyroidism out of which 135 (13.5%) were labelled with sub-clinical hypothyroidism and 7 (7.0%) with overt hypothyroidism [25]. The slight diversity in these figures could be a product of the difference in the diets between the two populations. Moreover, most of our sample population (231, 79.3%) constituted of women entitled to free health care and checkups.

In our study 60 women (20.5%) were hyperthyroid, out of which 12 women (4.10%) were classified as overt hyperthyroid and 48 women (16.4%) as sub-clinically hyperthyroid. Previous data from the general Pakistani population showed the prevalence rate of hyperthyroidism as 5.1%, whilst subclinical hyperthyroidism was 5.8%, with it being more predominant in the female population. These values could be attributed to the physiological changes in the levels of thyroid hormones (TSH, FT4, FT3) during pregnancy. Furthermore, thyrotoxicosis during the first trimester of pregnancy is reportedly higher in Asian populations compared to their European counterparts [26]. A Chinese study also showed an initial Increase in FT4 during the first trimester, along with an initial suppressed TSH. The greatest suppression of TSH occurred during 9 ± 13 weeks, when FT4 concentrations were the highest [27]. This further emphasizes the need to measure FT4 along with TSH to accurately assess the disease status in these women and to prevent the risk of over or under-diagnosing patients.

A study done in the United Kingdom demonstrated that independent of age, the greater the serum TSH above 2mIU/l, the greater are the chances of the developing overt hypothyroidism for these individuals [28]. In our study, 60 (20.48%) women had TSH levels greater than 2mU/L, which could pose a potential risk of developing hypothyroidism, in this cohort, in the future.

A trimester-specific scale based on the Pakistani population needs to be developed for more accurate diagnosis and treatment of women in the region, as there are variations in ranges used in different parts of the world. Without a uniform tool adopted unanimously across the country, health treatment can greatly vary and can have devastating outcomes leading to a rise in poor pregnancy outcomes. A Danish study compared and analyzed two cohorts of pregnant women with the counter group’s thyroid reference ranges. Their results showed a misdiagnosis in thyroid status, thus limiting the role of previously published thyroid reference ranges [29]. Race has been shown to be a major influencing factor, as studies done in china have showed a variation in TSH levels and FT4 and FT3 ranges [27].

Recent consensus guidelines do not advocate universal thyroid function screening during pregnancy in Pakistan but recommend testing high-risk pregnant women with a past or present history of a thyroid disorder, and patients with a first degree relative with thyroid disease or any other clinical indication of a thyroid disorder. However, there is a significant overlap of symptoms between the various groups of thyroid derangements and a strict clinical diagnosis is not possible [30]. There is also a significant subset of the population residing in rural areas without adequate antenatal check up facilities and the inability to afford access to healthcare facilities, which can exclude many women with potential thyroid dysfunction. Under these circumstances, we feel there could be a need for more vigorous thyroid screening in the area in order to accurately determine the disease burden and effectively manage patients.

Limitations

One of the limitations of our study was a small sample size. The outcomes of the pregnancy could also not be followed up on and hence a a link between TSH and FT4 levels with pregnancy outcomes could not be determined . A convenience sampling technique was used for this study instead of random sampling. Due to the limitation of funds, serum T3, thyroid autoantibodies (antibodies to thyroid peroxidase and TSH receptor antibodies) and serum iodine levels could not be assessed. It is suggested, to future researchers, to conduct a more comprehensive study to determine trimester specific thyroid hormone levels, for the Pakistani population. 

## Conclusions

There is a need for adequate monitoring and subsequent thyroxine supplementation during the first trimester of pregnancy in order to prevent maternal and fetal complications during this time, as we believe that thyroid dysfunction is being underdiagnosed. There is also a need for the development of trimester specific reference ranges for the local Pakistani population to further improve maternal healthcare. The causes of maternal hypothyroidism are several and need to be further investigated.
